# Antimicrobial susceptibility of bacterial uropathogens in a South African regional hospital

**DOI:** 10.4102/ajlm.v12i1.1920

**Published:** 2023-03-03

**Authors:** Alicia Naidoo, Afsana Kajee, Nomonde R. Mvelase, Khine Swe Swe-Han

**Affiliations:** 1 Department of Medical Microbiology, RK Khan Laboratory, National Health Laboratory Service, Durban, South Africa; 2 Department of Medical Microbiology, School of Laboratory Medicine and Medical Sciences, College of Health Sciences, University of KwaZulu-Natal, Durban, South Africa; 3 Department of Medical Microbiology, Inkosi Albert Luthuli Central Hospital, National Health Laboratory Services, Durban, South Africa

**Keywords:** antimicrobial susceptibility patterns, uropathogens, urinary pathogens, antibiotic resistance, urinary oral treatment

## Abstract

**Background:**

Urinary tract infections are common bacterial infections affecting millions worldwide. Although treatment options for urinary tract infections are well established, with ciprofloxacin long considered one of the antibiotics of choice, increasing antibiotic resistance may delay the initiation of appropriate therapy. While this increase in antimicrobial resistance has been demonstrated in multiple studies around the world, there is a dearth of information from developing countries.

**Objective:**

This study aimed to describe the antimicrobial susceptibility patterns of commonly isolated bacterial uropathogens in a South African hospital.

**Methods:**

Antimicrobial susceptibility data of isolates obtained from urine specimens at the RK Khan Hospital, a regional hospital in KwaZulu-Natal, South Africa, between January 2018 and December 2020 were retrieved from the hospital’s laboratory information system and analysed to determine the differences in resistance rates between the most frequently isolated bacterial uropathogens.

**Results:**

Of the 3048 bacterial urinary pathogens isolated between 2018 and 2020, *Escherichia coli* (1603; 53%) was the most common, followed by *Klebsiella* spp. (437; 14%). Both *E. coli* and *Klebsiella* spp. showed high rates of resistance to amoxicillin/clavulanic acid (29.8% and 42.3%) and ciprofloxacin (37.7% and 30.4%). Nitrofurantoin resistance was low among *E. coli* (6.2%) but high among *Klebsiella* spp. (61.3%).

**Conclusion:**

*E. coli* and *Klebsiella* spp. in this study were highly resistant to amoxicillin/clavulanic acid and ciprofloxacin, two of the frequently prescribed oral treatment options.

**What this study adds:**

This study highlights the importance of regular local antimicrobial resistance surveillance to inform appropriate empiric therapy.

## Introduction

Although the urinary tract has multiple mechanisms in place to keep it sterile, urinary tract infections (UTIs) are one of the most common bacterial infections affecting nearly 150 million people worldwide and amounting to more than $6 billion United States dollars in healthcare costs, with approximately 95% of all UTIs occurring because of periurethral contamination by enteric uropathogens.^1,2^ International studies indicate that due to the high incidence of UTIs and the widespread practice of empirical treatment, the rate of antibiotic prescriptions is also increasing.^3^ As antibiotic use is known to be the main driver of the evolution of resistance, the increasing use of antibiotics in the treatment of UTIs has contributed to the alarming increase in antimicrobial resistance among frequently isolated urinary pathogens, thus further limiting treatment options, particularly with orally administered drugs.^2,4,5,6^

*Escherichia coli, Klebsiella* spp., and *Enterococcus* spp., which are all members of the normal gut flora, are the most typically isolated organisms from urine, with *E. coli* being the most frequently isolated.^7,8^ Empiric treatment options for UTIs are well established globally, and these include ciprofloxacin, amoxicillin/clavulanic acid, nitrofurantoin, fosfomycin, and cephalosporins.^9,10^ However, studies conducted locally and internationally demonstrate that an increase in the use of most of these drugs has caused a surge in antimicrobial resistance and a concomitant increase in treatment failures.^2,11^ This exerts greater pressure on an already overburdened healthcare system. Antimicrobial resistance surveillance studies thus need to be conducted regularly to identify bacterial uropathogens and their antimicrobial susceptibility patterns to guide the empiric treatment of UTIs, which is a crucial part of antimicrobial stewardship. Therefore, this study aimed to determine the prevalence of bacterial uropathogens and their antimicrobial susceptibility patterns to assist antimicrobial stewardship in a regional hospital in KwaZulu-Natal, South Africa.

## Methods

### Ethical considerations

Ethics approval was granted by the University of KwaZulu-Natal Biomedical Research Ethics Committee, with permit number BREC/00001578/2020. Informed patient consent was not required by the University of KwaZulu-Natal Ethics Committee as this was a retrospective study and there was no patient interaction. Patient privacy and confidentiality of data were protected in accordance with the Declaration of Helsinki.

### Study design and setting

A retrospective study was performed by retrieving three years of laboratory data from 01 January 2018 to 31 December 2020 at the National Health Laboratory Services, RK Khan Hospital, Durban, KwaZulu-Natal, South Africa. The 543-bed hospital ranks as both a regional and district hospital and serves many internal and external clinics.

Data, which included patient location (inpatient and outpatient) and isolated uropathogens, were collected from the laboratory information system and captured onto a Microsoft Excel 365 spreadsheet, version 2205 (Microsoft Corporation, Redmond, Washington, United States). Interpreted antimicrobial susceptibility data (‘resistant’, ‘intermediate’, ‘sensitive’) for the retrieved isolates were also extracted from the laboratory information system.

#### Inclusion criteria

Data on all positive urine bacterial cultures processed between January 2018 and December 2020 were retrieved from the laboratory information system. We then determined the distribution of bacterial uropathogens and analysed the antimicrobial susceptibility patterns of the two most common Enterobacterales, namely *E. coli* and *Klebsiella* spp.

Considering the study setting and the current literature, *Enterococcus faecalis, Enterococcus faecium,* Group B *Streptococcus,* and *Staphylococcus* spp. were also chosen for analysis as they are the most common Gram-positive bacterial causes of UTI. Antibiotics analysed in the study were based on the recommended empiric therapy as per the South African Standard Treatment Guideline.^9^

#### Exclusion criteria

As this study was on bacterial uropathogens, all data on yeasts were excluded. To exclude duplicate entries, we used the following patient variables: patient name, surname, date of birth, and organism name. The organism name was included to remove duplicate entries from the same patient with the same organism.

### Laboratory analysis

Urine specimens were processed according to the laboratory’s routine standard operating procedure. Bacterial identification and antimicrobial susceptibility testing were carried out using the VITEK^®^ 2 automated system (Biomerieux, Marcy l’Ètoile, Rhône-Alpes, France). As cephalothin was not part of the antibiotics tested on the VITEK^®^ 2 N255 card, cephalothin susceptibility was analysed using the Kirby Bauer disk diffusion method. For Gram-negative bacteria, the antibiotics analysed were amikacin, amoxicillin/clavulanic acid, cefotaxime or ceftriaxone, ceftazidime, cephalothin, ciprofloxacin, gentamicin, meropenem, nitrofurantoin, piperacillin/tazobactam and trimethoprim/sulfamethoxazole. For the Gram-positive bacteria, the antibiotics analysed include ampicillin, cloxacillin, penicillin, and vancomycin. Results for cefotaxime and ceftriaxone were reported together as they have the same mechanism of action and resistance patterns and were therefore used interchangeably at RK Khan Hospital. All antimicrobial susceptibility results were interpreted using the Clinical and Laboratory Standards Institute guidelines.^12^

An extended-spectrum beta-lactamase producer was defined as an organism that was resistant to all cephalosporins, while carbapenem-resistant Enterobacterales were defined as isolates that were resistant to any carbapenem.

### Data analysis

We determined the antimicrobial resistance rates of the most commonly isolated Gram-negative (*E. coli* and *Klebsiella* spp.) and Gram-positive bacteria (*E. faecalis, E. faecium*, Group B *Streptococcus,* and *Staphylococcus* spp.). Additionally, for *E. coli*, we compared the antibiotic resistance rates between strains obtained from inpatients and outpatients and analysed the trends in antibiotic resistance rates over the three-year study period (2018–2020).

We also determined the statistical significance of differences observed in the resistance rates between *E. coli* and *Klebsiella* spp., as well as differences in the resistance rates of *E. coli* strains isolated from inpatients and outpatients. These statistical comparisons were done using the Chi-square test, where a *p*-value less than 0.05 was considered statistically significant. The *p*-values for the pairwise comparisons were adjusted using the Bonferroni correction method (R Statistical Computing Software, version 3.6.3, R Core Team, Auckland, New Zealand).

## Results

From January 2018 to December 2020, the National Health Laboratory Services RK Khan Laboratory analysed 3804 urine samples from patients with suspected UTIs. After removing duplicate data and data on yeasts, data on a total of 3048 bacterial isolates were included in this study.

**FIGURE 1:**
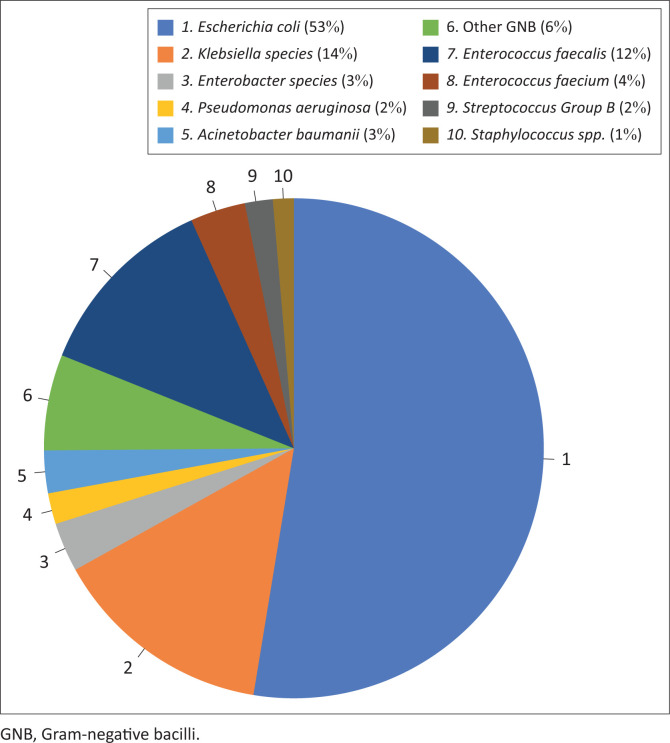
Distribution of the 3048 bacterial urinary pathogens isolated at the RK Khan Hospital, Durban, KwaZulu-Natal, South Africa, January 2018 – December 2020.

*Escherichia coli* (*n* = 1603; 53%) was the most frequent urinary pathogen isolated, followed by *Klebsiella* spp. (*n* = 437; 14%), including 398 (13%) *Klebsiella pneumoniae* and 39 (1%) *Klebsiella oxytoca* (Figure 1). *Enterobacter* spp. (*n* = 97; 3.2%) (including 82 [2.7%] *Enterobacter cloacae* and 15 [0.5%] *Enterobacter aerogenes*), *Pseudomonas aeruginosa* (*n* = 61; 2%), *Acinetobacter baumannii* (*n* = 84; 3%), and other Gram-negative bacilli (*n* = 189; 6%) made up a further 14%. *Enterococcus faecalis* (*n* = 372; 12%) was the most frequent Gram-positive organism isolated, followed by *E. faecium* (*n* = 109; 4%), Group B *Streptococcus* (*n* = 55; 2%), and *Staphylococcus* spp. (*n* = 41; 1%).

As *E. coli* and *Klebsiella* spp. made up the majority of the Gram-negative bacilli isolated, it was important to determine their antimicrobial resistance rates. The total number of cephalothin susceptibility results differed from the other antibiotics because cephalothin susceptibility was determined manually and the result was not always entered into the laboratory information system. Similarly, the total number of results was different for each antimicrobial tested due to missing results linked to random terminations that can sometimes occur during automated antimicrobial susceptibility testing with the VITEK^®^ 2 instrument.

**FIGURE 2:**
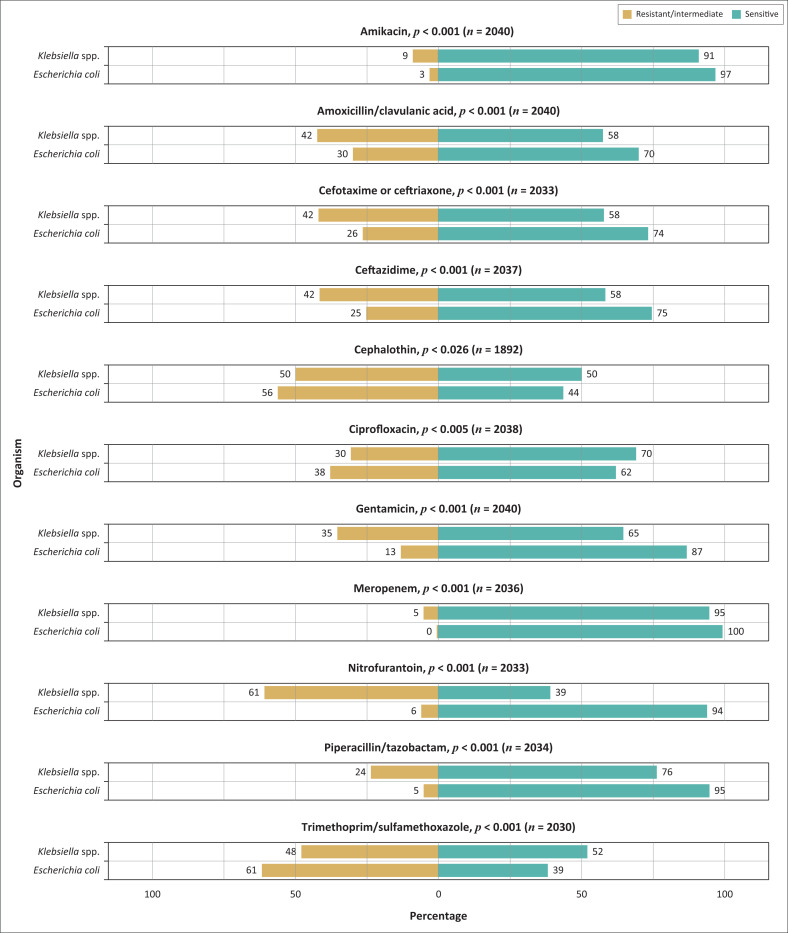
Antimicrobial susceptibility rates of *E. coli* and *Klebsiella* spp. isolated from patients with suspected urinary tract infections at the RK Khan Hospital, Durban, KwaZulu-Natal, South Africa, January 2018 – December 2020. The total number of isolates is different for each antimicrobial due to missing results linked to random terminations that can sometimes occur during automated antimicrobial susceptibility testing with the VITEK^®^ 2 instrument.

*E. coli* demonstrated low levels of resistance to meropenem (0.56%; *n* = 9), amikacin (2.9%; *n* = 47), nitrofurantoin (6.2%; *n* = 99) and piperacillin/tazobactam (5.3%; *n* = 85) (Figure 2). A very high level of resistance was noted in recommended oral treatment options, namely trimethoprim/sulfamethoxazole (61%; *n* = 988), ciprofloxacin (38%; *n* = 605) and amoxicillin/clavulanic acid (30%; *n* = 478). Similarly, *Klebsiella* spp. strains were highly resistant to nitrofurantoin (61%; *n* = 268), trimethoprim/sulfamethoxazole (48%; *n* = 208), amoxicillin/clavulanic acid (42%; *n* = 185), and ciprofloxacin (30%; *n* = 133). Twenty-six percent of *E. coli* (*n* = 424) isolates were extended-spectrum beta-lactamase producers and 0.6% (*n* = 9) were carbapenem-resistant. Among the *Klebsiella* spp. isolates, 41.8% (*n* = 184) were extended-spectrum beta-lactamase producers and 5% (*n* = 22) were carbapenem-resistant.

Overall, *Klebsiella* spp. was significantly more resistant than *E. coli* to all the tested antimicrobials except cephalothin (*E. coli –* 56% vs *Klebsiella* spp. – 50%), ciprofloxacin (38% vs 30%), and trimethoprim/sulfamethoxazole (61% vs 48%). For all comparisons except cephalothin (*p* = 0.03) and ciprofloxacin (*p* = 0.005), the *p*-values were less than 0.001.

Among the Gram-positive bacteria, *E. faecium* isolates showed high levels of resistance to both ampicillin (83.5%; *n* = 91) and penicillin (83.5%; *n* = 91) (Table 1). Conversely, *E. faecalis* isolates had low rates of resistance to ampicillin (0.8%; *n* = 3) and penicillin (2.4%; *n* = 9). In addition, the *Staphylococcus* spp. isolates had high resistance rates to ampicillin (*n* = 38; 92.7%) and penicillin (*n* = 38; 92.7%). Vancomycin was the most effective antibiotic against *E. faecium* (97.2% susceptibility) and *Staphylococcus* spp. (100% susceptibility), while ampicillin and penicillin were most effective against Group B *Streptococci* (100% susceptibility).

**TABLE 1:** Antimicrobial susceptibility patterns of commonly isolated urinary Gram-positive cocci at the RK Khan Hospital, Durban, KwaZulu-Natal, South Africa, January 2018 – December 2020.

Antibiotic	Organisms							
	*Enterococcus faecalis* (*N* = 372)		*Enterococcus faecium* (*N* = 109)		Group B *Streptococci* (*N* = 55)		*Staphylococcus* spp. (*N* = 41)	
	*n*	%	*n*	%	*n*	%	*n*	%
Ampicillin								
Susceptible	369	99.2	18	16.5	55	100	3	7.3
Resistant/intermediate	3	0.8	91	83.5	0	0	38	92.7
Penicillin								
Susceptible	363	97.6	18	16.5	55	100	3	7.3
Resistant/intermediate	9	2.4	91	83.5	0	0	38	92.7
Vancomycin								
Susceptible	367	N/A	-	97.2	ND	ND	41	100
Resistant/intermediate	5	N/A	-	2.8	ND	ND	0	0
Cloxacillin								
Susceptible	N/A	N/A	N/A	N/A	N/A	ND	35	85.3
Resistant/intermediate	N/A	N/A	N/A	N/A	N/A	ND	6	14.6

ND, not done; N/A, not applicable.

For analysis of inpatient and outpatient data, only *E. coli* was studied as it constituted more than half of the bacterial pathogens. Five hundred and eleven *E. coli* isolates (32%) were isolated from inpatients, while 1092 (68%) were from outpatients (Table 2). Low level of resistance was observed in both inpatient and outpatient wards for meropenem (0.8%; *n* = 4 and 0.1%; *n* = 1), amikacin (3.1%; *n* = 16 and 2.8%; *n* = 31), piperacillin/tazobactam (5.5%; *n* = 28 and 4.7%; *n* = 51) and nitrofurantoin (6.3%; *n* = 32 and 5.5%; *n* = 60). However, there were high rates of resistance to trimethoprim/sulfamethoxazole (inpatients: 69.7%; *n* = 355, outpatients: 57.6%; *n* = 625), ciprofloxacin (inpatients: 34.7%; *n* = 177, outpatients: 39%; *n* = 426), and amoxicillin/clavulanic acid (inpatients: 32.9%; *n* = 168, outpatients: 28.4%; *n* = 310). The prevalence of extended-spectrum beta-lactamases and carbapenem resistance in *E. coli* was higher among inpatients (31.0%; *n* = 158 and 0.8%; *n* = 4) compared to outpatients (24%; *n* = 261 and 0.1%; *n* = 1).

**TABLE 2:** Antimicrobial susceptibility patterns of *E. coli* isolated from inpatient and outpatient wards at the RK Khan Hospital, Durban, KwaZulu-Natal, South Africa, January 2018 – December 2020

Antimicrobial agents	Inpatients			Outpatients			*p*
	Total number of isolates	Resistant/intermediate		Total number of isolates	Resistant/intermediate		
	
	*N*	*n*	%	*N*	*n*	%	
Amikacin	511	16	3.1	1092	31	2.8	0.75
Amoxicillin/clavulanic acid	511	168	32.9	1092	310	28.4	0.07
Cefotaxime or ceftriaxone	510	158	31.0	1088	261	24.0	0.003
Ceftazidime	511	149	29.2	1090	254	23.3	0.012
Cephalothin	464	265	57.1	1092	573	52.5	0.57
Ciprofloxacin	510	177	34.7	1092	426	39.0	0.1
Gentamicin	511	78	15.3	1092	131	12.0	0.07
Meropenem	511	4	0.8	1088	1	0.1	0.02
Nitrofurantoin	509	32	6.3	1087	60	5.5	0.54
Piperacillin/tazobactam	511	28	5.5	1086	51	4.7	0.50
Trimethoprim/ sulfamethoxazole	509	355	69.7	1086	625	57.6	< 0.001

Note: The total number of isolates is different for each antimicrobial due to missing results linked to random terminations that can sometimes occur during automated antimicrobial susceptibility testing with the VITEK^®^ 2 instrument.

There were no statistically significant differences in resistance rates to recommended oral antibiotics, namely amoxicillin/clavulanic acid (*p* = 0.07), ciprofloxacin (*p* = 0.10) and nitrofurantoin (*p* = 0.54) between the *E. coli* isolates from inpatients and outpatients. However, for trimethoprim/sulfamethoxazole, *E. coli* isolates from inpatient wards were significantly more resistant than those from outpatient wards (*p* < 0.001).

**FIGURE 3:**
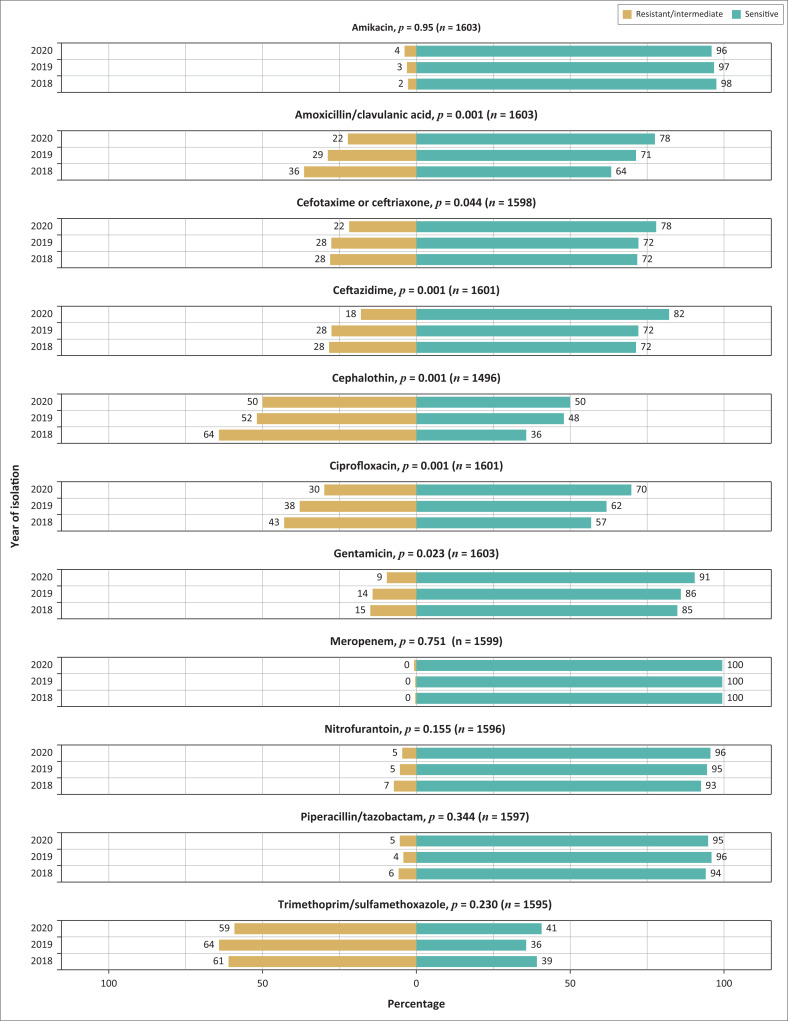
Antimicrobial susceptibility trends of *E. coli* isolated from patients with suspected urinary tract infections at the RK Khan Hospital, Durban, KwaZulu-Natal, South Africa, January 2018 – December 2020. The total number of isolates is different for each antimicrobial due to missing results linked to random terminations that can sometimes occur during automated antimicrobial susceptibility testing with the VITEK^®^ 2 instrument.

Of the 1603 *E. coli* strains isolated between 2018 and 2020, 625 (39.1%) strains were isolated in 2018, 539 (33.6%) were isolated in 2019, and 439 (27.4%) were isolated in 2020, demonstrating slight decreases in prevalence over the study period (Figure 3). Amikacin and meropenem resistance rates, while low, increased slightly between 2018 (2.4% and 0.5%) and 2020 (3.6% and 0.9%). Conversely, the *E. coli* isolates had higher resistance rates to the more commonly prescribed oral antibiotics, but these rates decreased steadily between 2018 and 2020 (cephalothin – 63.9% vs 49.8%; ciprofloxacin – 42.9% vs 30.4%; amoxicillin/clavulanic acid – 36.6% vs 22.4%).

Over the three years (2018–2020), there were no statistically significant differences in resistance rates to amikacin (*p* = 0.50), meropenem (*p* = 0.75), nitrofurantoin (*p* = 0.16), piperacillin/tazobactam (*p* = 0.34), and trimethoprim/sulfamethoxazole (*p* = 0.2). In contrast, resistance rates to amoxicillin/clavulanic acid, ceftazidime, cephalothin and ciprofloxacin decreased significantly between 2018 and 2020 (*p* < 0.001). The *E. coli* isolates also demonstrated a significant decrease in resistance to cefotaxime or ceftriaxone (*p* = 0.04) and gentamicin (*p* = 0.02).

## Discussion

In this study, we found that *E. coli* was the most frequently isolated uropathogen at the RK Khan Hospital, Durban, KwaZulu-Natal, South Africa, between 2018 and 2020, followed by *Klebsiella* spp. and *E. faecalis*. We also found that the commonly identified urinary pathogens had relatively high resistance rates to the widely administered antibiotics for UTI treatment.

Our findings are consistent with those of previous local and international studies.^13,14^ A study conducted among patients with community-acquired UTIs in India showed that *E. coli* (55.1%), *E. faecalis* (15.8%), and *K. pneumoniae* (13.7%) were the most prevalent uropathogens isolated.^13^ Similarly, a six-year (2011–2016) study conducted in the obstetric departments of six public-sector hospitals in Durban, KwaZulu-Natal, South Africa, showed that *E. coli* (54.2%) was the most common uropathogen, followed by *K. pneumoniae* (12.9%).^14^

In 2010, the Infectious Diseases Society of America and the European Society of Microbiology and Infectious Disease published a clinical practice guideline outlining their recommendations for the treatment of uncomplicated UTIs, with nitrofurantoin, fosfomycin, trimethoprim/sulfamethoxazole, and ciprofloxacin recommended as the drugs of choice.^6^ Similarly, in the South African Standard Treatment Guideline, published in 2019, fosfomycin, ciprofloxacin, amoxicillin/clavulanic acid, and nitrofurantoin are also considered as empiric oral treatment options for UTIs.^9^ In their guidelines, both the Infectious Diseases Society of America and the European Society of Microbiology and Infectious Disease recommend that ciprofloxacin should be used as an empiric antibiotic therapy provided that resistance rates remain below 10%.^6^ It was therefore disconcerting to discover that, in our study, both *E. coli* and *Klebsiella* spp. showed high resistance rates against ciprofloxacin and amoxicillin/clavulanic acid. Over 30% of the *E. coli* and *Klebsiella* spp. isolates were resistant to ciprofloxacin and amoxicillin/clavulanic acid. In contrast, while only 6.2% of *E. coli* were resistant to nitrofurantoin, *Klebsiella* spp. showed a high rate (61.3%) of nitrofurantoin resistance. Although nitrofurantoin is still an option for treating UTIs caused by *E. coli*, our study suggests that it is ineffective against the majority of *Klebsiella* spp. infections, leaving physicians with limited empiric oral therapy alternatives.

Similar to our findings, in a 9-year (2011–2019) study conducted in Europe,^15^ resistance rates to ciprofloxacin and amoxicillin/clavulanic acid exceeded the 10% threshold for both *E. coli* (42.1% and 14.3%) and *Klebsiella* spp. (68.7% and 38.8%). Although the *E. coli* nitrofurantoin resistance rate (4.8%) in our study was below this 10% threshold, the resistance rate for *Klebsiella* spp. (46.0%) far exceeded the threshold. These high rates of resistance to recommended oral empiric therapy are of major concern as this could lead to increased treatment failure rates.

As our study was laboratory-based and the relevant information was not available on the laboratory information system, we were unable to distinguish between community-acquired and hospital-acquired UTIs. We, therefore, examined the antimicrobial patterns of *E. coli* isolates from the inpatient wards versus the outpatient wards. Although resistance rates were generally higher (between 1% and 12% difference) among inpatients than outpatients, these differences were not statistically significant, except for trimethoprim/sulfamethoxazole. A Japanese study done in 2020 showed that the differences in *E. coli* resistance rates between hospital-acquired and community-acquired UTIs were between 5% and 10%.^16^

According to the South African public healthcare system, new patients do not directly present to hospitals. Instead, they are initially seen at local clinics and only referred to hospitals if required.^9^ As a result, patients who are seen at hospitals are either known hospital patients with chronic diseases or patients who need a higher level of care than the primary healthcare offered in the clinics. This may explain the high resistance rates in frequently isolated urinary bacteria in our study. It is however interesting to note that in a local study conducted in KwaZulu-Natal, South Africa, between 2011 and 2016, lower levels of resistance to recommended empiric oral antimicrobials were reported among *E. coli* isolates from pregnant, but otherwise healthy, individuals who were generally not on antimicrobial therapy.^14^ Despite these rates being lower than those of our study, they are still above the empirical antibiotic resistance threshold of 10% recommended by the Infectious Diseases Society of America and the European Society of Microbiology and Infectious Disease.

### Limitations

Because this was a retrospective study, the reliability of the data presented depended on the accuracy of the recording of the initial patient results. The resistance rates in this study may have been overrepresented as clinicians are unlikely to request susceptibility testing for patients that respond to empiric therapy. Also, due to a lack of clinical information, we were unable to determine the difference between hospital-acquired and community-acquired infections.

### Conclusion

Our study revealed that *E. coli* was the most predominant urinary pathogen isolated and that antimicrobial resistance levels in commonly isolated uropathogens remain elevated, especially against frequently prescribed oral antimicrobials. Our findings indicate that the current empiric therapy used in the treatment of UTIs would fail in a large proportion of affected individuals, thus highlighting the need for more research to evaluate alternate oral drugs such as fosfomycin, which is not in use in the public sector despite being recommended in the South African Standard Treatment Guideline.^9^
